# Monoplane 3D reconstruction of mapping ablation catheters: a feasibility study

**DOI:** 10.2349/biij.6.2.e17

**Published:** 2010-04-01

**Authors:** P Fallavollita

**Affiliations:** School of Computing, Queen’s University, Kingston, Ontario, Canada

**Keywords:** 3D reconstruction, monoplane, C-arm fluoroscopy, catheter ablation, cardiac arrhythmias

## Abstract

**Purpose:**

Radiofrequency (RF) catheter ablation has transformed treatment for arrhythmias and has become first-line therapy for some tachycardias. The precise localization of the arrhythmogenic site and the positioning of the RF catheter over that site are problematic: they can impair the efficiency of the procedure and are time consuming (several hours). This study evaluates the feasibility of using only single plane C-arm images in order to estimate the 3D coordinates of RF catheter electrodes in a cardiac phase.

**Materials and methods:**

The method makes use of *a priori* 3D model of the RF mapping catheter assuming rigid body motion equations in order to estimate the 3D locations of the catheter tip-electrodes in single view C-arm fluoroscopy images. Validation is performed on both synthetic and clinical data using computer simulation models. The authors' monoplane reconstruction algorithm is applied to a 3D helix mimicking the shape of a catheter and undergoing solely rigid motion. Similarly, the authors test the feasibility of recovering nonrigid motion by applying their method on true 3D coordinates of 13 ventricular markers from a sheep’s ventricle.

**Results:**

The results of this study showed that the proposed monoplane algorithm recovers rigid motion adequately when using the spatial positions of a catheter in six consecutive C-arm image frames yielding maximum 3D root mean squares errors of 4.3 mm. On the other hand, the suggested algorithm did not recover nonrigid motion precisely as suggested by a maximum 3D root mean square value of 8 mm.

**Conclusion:**

Since RF catheter electrodes are rigid structures, the authors conclude that there is promise in recovering the 3D coordinates of the electrodes when making use of only single view images. Future work will involve adding nonrigid motion equations to their algorithm, which will then be applied to actual clinical data.

## INTRODUCTION

In 2005, the incidence of sudden cardiac death (SCD) in the United States was about 290,000 cases per year [1]. Left ventricular dysfunction, such as ventricular tachycardia (VT), is currently the best available predictor for SCD [2]. Severe disorders of the heart rhythm that can cause sudden cardiac death or morbidity, can be treated by radio-frequency (RF) catheter ablation, which consists of inserting a catheter inside the heart, near the area from which the abnormal cardiac electrical activity originates, and delivering RF current through the catheter tip so as to ablate this arrhythmogenic area [3]. The precise localization of the arrhythmogenic site and positioning of the RF catheter at that site are problematic: they can impair the efficacy of the procedure, which can last many hours, especially for complex arrhythmias [3]. To shorten the duration of RF catheter ablation and increase its efficiency, commercial systems that provide a 3D color display of the cardiac electrical activation sequence during the arrhythmia have been proposed. These systems incorporate basket electrode arrays (*Constellation*, EPT Inc.), catheters with a balloon electrode array (*Ensite 3000*, Endocardial Solutions Inc.) and catheters with magnetic position detectors (*CARTO XP*, Biosense Webster Inc.). Lastly, a complete navigation and registration framework is available (*CartoMerge*, Biosense Webster Inc.).

The *CARTO XP* ablation mapping and navigation system provides real-time data on 3D, color-coded maps of the electrical activity of the heart. The *CARTO XP* system makes possible precise, real-time tracking of catheter location by using magnetic fields. The mapping catheter resembles a standard deflectable ablation catheter with a 4-mm tip and proximal 2-mm ring electrodes. The location sensors lie adjacent to the tip electrode, totally embedded within the catheter. The three location sensors are located orthogonally to each other. A locator pad is placed beneath the operating table and includes three coils that generate low magnetic fields, which decay as a function of the distance from their sources. When the catheter is moved within this magnetic field, signals received by the sensors are transmitted along the catheter shaft to the main processing unit so as to track its position in 3D. This approach enables tracking of the catheter independent of fluoroscopy [4].

The *EnSite 3000* system provides electrophysiologists with a real-time, virtual image of the electrical activity of the heart without contacting the heart’s surface. The electrode array consists of a small balloon around which are woven 64 insulated wires with a single break in their insulation, producing 64 unipolar electrodes. This array is mounted at the end of a catheter, which is introduced in the cardiac chamber to be investigated. When placed in the chamber, the small balloon is partially inflated. The balloon does not fill the chamber and the electrodes do not make contact with the cardiac wall (which is why this approach is called non-contact). A multi-channel amplifier and computer workstation processes the raw far-field electrographic data and displays 3D anatomical information [5].

Simultaneous mapping of multiple points is performed using a 64-lead basket *Constellation* catheter that can be deployed percutaneously. Current designs of basket arrays consist of a series of equally spaced electrodes mounted on eight flexible splines. Each spline contains eight 1.5 mm electrodes with 3-mm spacing. The catheter is introduced percutaneously through a sheath into the chamber. By pulling back the sheath, the splines deploy and are apposed against the endocardium. The basket catheter is connected via amplifiers to the mapping system. The signals are filtered from 30 to 400 Hz. Detection of local activation is performed for each electrogram and 2D isochronal maps are generated but with no 3D display of the anatomy [5].

All these systems are costly [6]. The first two can map the cardiac activation sequence using data recorded during a single beat whereas the *CARTO* system relies on data recorded point-by-point during numerous beats, which implies that the arrhythmia must remain stable during the procedure.

Recently, the authors have proposed a more affordable fluoroscopic navigation system by obtaining local activation times from a roving mapping catheter, when treating VT, whose positions are computed from biplane fluoroscopic projections, and by superimposing the isochronal map depicting the cardiac electrical activation sequence over the fluoroscopic image of the heart [3]. As biplane C-arm fluoroscopes are not commonly available in hospitals, the authors attempted to estimate the depth of the tip-electrode of a mapping catheter using only a single image. The final results yielded depth estimations of about 10 mm. Due to this large error, continued research on estimating the 3D coordinates of the tip electrode using only a single-view fluoroscopic sequence is primordial.

The focus of this paper establishes two significant modifications to the previous work. First, the authors will consider a full perspective camera model instead of orthographic projection so as to create a more precise 3D geometry of the mapping catheter and the electrodes in it. Second, they propose to use *a priori* 3D information of the mapping ablation catheter positions in order to estimate its depth on C-arm fluoroscopy images using only single-plane image sequences. The authors will exploit the spatio-temporal information in the C-arm images to compensate for the unknown z-value of the tip-electrode. This intuition of exploiting multiple single-view images should lead to a more precise depth estimate of the catheter. The authors emphasize that this work is a feasibility study and, therefore, computer simulations depicting both rigid and non-rigid movement of the catheter will be used for assessment of their proposed methodology. Lastly, to their knowledge, this analysis is a first of its kind for single-view 3D reconstruction and depth estimation for the purpose of assisting catheter ablation procedures.

## METHODOLOGY

### Full perspective camera model

**Figure 1:**
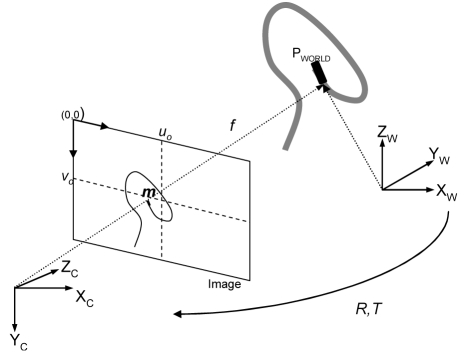
The perspective camera model. Any 3D world point can be projected onto a 2D plane and its coordinates would be (*u, v*) pixels. The camera model is taken from the Epipolar Geometry Toolbox (adapted from [3]). Image resolution would be equal to C-arm intensifier size divided by image size in pixels.

Figure 1 shows the full perspective camera model that will be used for the 3D reconstruction problem [7]. If the authors define a 3D point *P_world_*= [X Y Z 1]^T^ in the world coordinate system, then its 2D projection in an image, *m*= [u v 1]^T^, is achieved by constructing a projection matrix:

(1) $$\begin{array}{c} P_{mat}=\;\left[\begin{array}{ccc} kf & 0 & u_{o}\\ 0 & kf & v_{o}\\ 0 & 0 & 1 \end{array}\right]\;\times \;\left[\begin{array}{cccc} r_{11} & r_{12} & r_{13} & t_{x}\\ r_{21} & r_{22} & r_{23} & t_{y}\\ r_{31} & r_{32} & r_{33} & t_{z} \end{array}\right]\\ \;m=\;P_{mat}P_{world} \end{array}$$

The intrinsic matrix of size [3x3], contains the pixel coordinates of the image center, also known as the principal point (*u_o_, v_o_*), the scaling factor *k*, which defines the number of pixels per unit distance in image coordinates, and the focal length *f* of the camera (in meters). The extrinsic matrix of size [3x4] is identified by the transformation needed to align the world coordinate system to the camera coordinate system. This means that a translation vector, *t*, and a rotation matrix, *R*, need to be found in order to align the corresponding axis of the two reference frames. Lastly, image resolution (usually mm/pixel) is calculated from the imaging intensifier size divided by the actual size of the image in pixels.

### Orthographic and weak perspective camera model

An orthographic camera is one that uses parallel projection to generate a 2D image of a 3D object. The image plane is perpendicular to the viewing direction. Parallel projections are less realistic than full perspective projections, however, they have the advantage that parallel lines remain parallel in the projection, and distances are not distorted by perspective foreshortening. The parallel projection matrix is given by:

(2) $$P_{affine}=\left[\begin{array}{cccc} k*r_{11} & k*r_{12} & k*r_{13} & (k*t_{x})\;+u_{o}\\ k*r_{21} & k*r_{22} & k*r_{23} & (k*t_{y})\;+\;v_{o}\\ 0 & 0 & 0 & 1 \end{array}\right]$$

The weak perspective camera is an approximation of the full perspective camera, with individual depth points *Z_i_* replaced by an average depth *Z_avg_*. The authors define the average depth, *Z_avg_* as being located at the centroid of the cloud of 3D points in the world coordinate system. The weak perspective projection matrix is given by:

(3) $$\begin{array}{c} P_{weak}=\left[\begin{array}{cccc} f*k*r_{11} & f*k*r_{12} & f*k*r_{13} & \left(f*k*t_{x})\;+\;(u_{o}*Z_{avg}\right)\\ f*k*r_{21} & f*k*r_{22} & f*k*r_{23} & \left(f*k*t_{y})\;+\;(v_{o}*Z_{avg}\right)\\ 0 & 0 & 0 & Z_{avg} \end{array}\right]\\ where\;Z_{avg}=\;([r_{31}\;r_{32}\;r_{33}]{}^{T}\times \;centroid)\;+\;t_{z} \end{array}$$

### A priori 3D monoplane algorithm

The authors first suppose that they have at their disposal a set of *n* 3D ablation catheter electrode points (*X0_n_, Y0_n_, Z0_n_*) at time *t* = 0 obtained from biplane fluoroscopic data. They also suppose that this first time instant reflects the diastolic cardiac phase. In this phase, the ventricles are filled with blood and the heart motion is at its smallest allowing for a more accurate 3D representation. The *a priori* coordinates are expressed in the camera reference frame in order to have a *Z*-direction corresponding to catheter electrode depth. The authors also have at their disposal the C-arm fluoroscope gantry parameters, which can be extracted from the image header DICOM files. These parameters enable them to construct a projection matrix for a specific viewing angle by using the full perspective camera model. They can now solve for the 3D displacements (*dx_i,i+1_, dy_i,i+1_, dz_i,i+1_*) between consecutive C-arm image frames beginning with the first image *i*=1. Expanding equation (1) and using an additional fluoroscopy image frame *i* =2, they obtain their first two equations as follows:

(4) $$\begin{array}{c} u_{2}=\;\frac{m_{1}(X0+dx_{12})+m_{2}(Y0+dy_{12})+m_{3}(Z0+dz_{12})+m_{4}}{m_{9}(X0+dx_{12})+m_{10}(Y0+dy_{12})+m_{11}(Z0+dz_{12})+m_{12}}\\ v_{2}=\;\frac{m_{5}(X0+dx_{12})+m_{6}(Y0+dy_{12})+m_{7}(Z0+dz_{12})+m_{8}}{m_{9}(X0+dx_{12})+m_{10}(Y0+dy_{12})+m_{11}(Z0+dz_{12})+m_{12}} \end{array}$$

Both equations describe the pixel coordinates in the second image (*u_2_, v_2_*) and the twelve coefficients *m_i=1:12_* are the values of the projection matrix. By adding an additional fluoroscopy image frame at time *i* = 3, we obtain two new equations with three additional unknowns in 3D:

(5) $$\begin{array}{c} u_{3}=\;\frac{m_{1}(X0+dx_{12}+dx_{23})+m_{2}(Y0+dy_{12}+dy_{23})+m_{3}(Z0+dz_{12}+dz_{23})+m_{4}}{m_{9}(X0+dx_{12}+dx_{23})+m_{10}(Y0+dy_{12}+dy_{23})+m_{11}(Z0+dz_{12}+dz_{23})+m_{12}}\\ v3=\;\frac{m_{5}(X0+dx_{12}+dx_{23})+m_{6}(Y0+dy_{12}+dy_{23})+m_{7}(Z0+dz_{12}+dz_{23})+m_{8}}{m_{9}(X0+dx_{12}+dx_{23})+m_{10}(Y0+dy_{12}+dy_{23})+m_{11}(Z0+dz_{12}+dz_{23})+m_{12}} \end{array}$$

The previous four equations take into account the spatial positions of a projected 3D world point on the acquired C-arm images. As the authors have four equations with six unknowns, they can extract two additional equations based on the fact that the Euclidean distances in pixels, *d*, between catheter electrode points in two consecutive images are known. It is to note that the distance between 3D points is not the same as the distance between their projected image points. Thus, they consider orthogonal projection estimations in this case and deem that this approximation is suitable enough for the proposed analysis. The authors arrive at the following two equations:

(6) $$\begin{array}{c} d_{12}{}^{2}=\sqrt{{\left(u_{2}-u_{1}\right)}^{2}+{\left(v_{2}-v_{1}\right)}^{2}}\;\\ d_{23}{}^{2}=\sqrt{{\left(u_{3}-u_{2}\right)}^{2}+{\left(v_{3}-v_{2}\right)}^{2}}\; \end{array}$$

They can now solve for the 3D displacements. A Levenberg-Marquardt optimization scheme [8] can be used here in order to solve for the unknown displacements. For the optimization scheme, initial approximations are a requirement to initialize the process. Hence, a suitable approximation for the displacements *dx* and *dy* can be obtained if the authors consider a parallel back projection of the 2D image points into the world coordinate system. As for the displacements *dz*, if they assume that the average depth of the catheter electrodes remains relatively constant in consecutive time frames (i.e. weak perspective camera model), then they can calculate the average depth of the 3D points (*X0, Y0, Z0*) in the second image (*t* = 1). This mean depth should be the same at time instants *i* =2, 3, etc., signifying that depths *dz* will be set to zero for the optimization scheme. However, for the sake of a more exhaustive analysis, they will also consider depth displacements *dz* ∈ [1-5] millimeters as well.

#### Single view algorithm motivation

A rationale use of the authors' algorithm would be to acquire biplane information at the beginning of the procedure and track catheter positions from monoplane fluoroscopy images during successive heart chamber mapping. They realize that they can select non-consecutive image frames as well, however, an accurate 3D reconstruction is only representative by taking into account all image frames. This does not diminish the overall effort of determining if and when the single-view algorithm fails and at what time instants it happens. For example, if the algorithm proves that single-plane reconstruction can be achieved reliably in the first 5 time instants and then fails, nothing impedes the authors to re-perform two-view reconstruction at that specific time in order to compensate for the failed results.

#### Evaluation

To validate the authors' proposed monoplane algorithm, they performed first rigid and non-rigid synthetic experimentation of structures. Authors in [9] modeled a cardiac intravascular (IVUS) transducer as a helix, and since a radio-frequency mapping catheter has the same tubular characteristics as the IVUS transducer, they represented it by a helix as well. They created a 3D helix in space and applied only rigid movement to it in a temporal fashion. The 3D points were re-projected in single-view 2D images. They also had at their disposal, 13 ventricle markers of a sheep with their temporal 3D coordinates as ground truth. These markers represent well the inherent non-rigid movement of the heart and should depict accurately the true movement of the heart. They projected these 3D markers on single-plane synthetic images as well and ran their monoplane algorithm.

## RESULTS AND DISCUSSION

### Computer simulations: rigid motion

The authors created a 3D helix containing 30 coordinate points so as to model the shape of a catheter. Then, they defined a biplane C-arm gantry setup that represented the posterior/anterior (PA) and left lateral (LAT) views of the heart. The focal length of the fluoroscopic system was set to a typical value of 100 cm and the helix location was set to 50 cm along the focal axis. The primary angles were equal to (90°, 0°), respectively, for the PA and LAT views, whereas the secondary angles were equal to (0°, 0°) for both views, respectively. The image sizes were set to [512x512] pixels and the C-arm intensifier size was chosen to be [178x178] mm. This allowed for a resolution 0.347 mm/pixel. They could now calculate two projection matrices and projected the 3D helix points on a first set of biplane images. These biplane images represent a first time instant at *t*=0. Subsequent biplane images were calculated by applying rigid movement on the 3D helix coordinates using the following rigid motion equation:

(7) $$X_{t=2}=\;R_{\theta \varphi \psi }\times X_{t=1}\;+\;[T_{x},T_{y},T_{z}]$$

**Figure 2:**
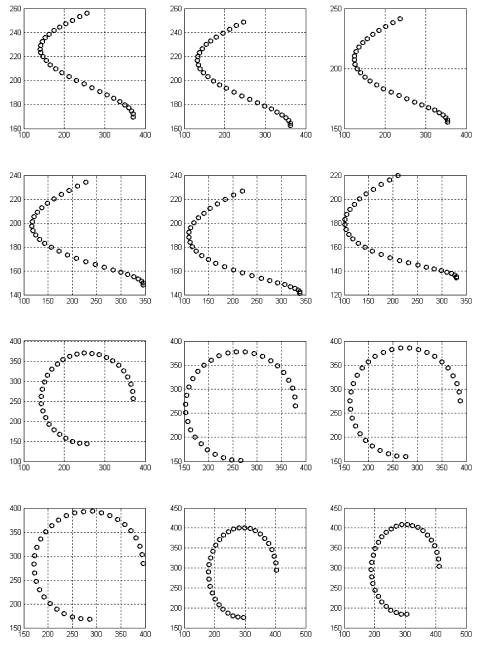
2D coordinates across six images using a 3D helix. In this case, 3Drotations across the three axis was set to 0.5 degrees, whereas 3D translations were set to 1.5mm in the X, Y and Z directions. (*Top*) Left Lateral view projections across six temporal images. The average interframe pixel distance was 6.5 pixels in both the x and y directions. (*Bottom*) Posterior/Anterior view projections. Interframe pixel displacements were on average 5 and 7 pixels in the x and y directions, respectively.

The 3D angles were set to *[θ, Φ, ϕ*] ∈ [0.5°, 0.5°, 0.5°] and the 3D translations were set to [*Tx, Ty, Tz*] ∈ [1, 1, 1] mm each. This accounted for average 2D interframe helix displacements in the images of (-6.82, -6.03) pixels in the left lateral view and (4.97, 7.12) pixels for the posterior/anterior views. These average displacements depict well a standard 15 fps acquisition rate for a typical C-arm procedure. A new set of biplane images and 3D helix points are obtained at *t*=1. In a similar fashion, equation (7) is re-applied to produce subsequent biplane data. Excluding the *a priori* biplane set at *t*=0, a total of five biplane datasets are generated. For good measure, they added error of up to 2 mm in coordinates. They tested their single-plane algorithm from three to six consecutive time instants (i.e. using three to six consecutive monoplane images). Figure 2 shows an example of the 2D projections of the helix using the specified gantry settings. The displacement between each image frame for the left lateral view was on average 6.5 pixels, whereas the displacement between each image frame for the posterior/anterior view was 5 and 7 pixels, respectively. Using orthographic projections for the approximations of *dx* and *dy* to solve their monoplane equations, they obtain approximations in the range of [1.7-2.4] millimeters using the intensifier size and image size values.

Table 1 shows the simulation results for rigid motion analysis on a helix. They observe that as the number of images used to optimize their monoplane equations increases, the overall reconstruction results deteriorate. This is expected as the uncertainty of landmark positions increases temporally in a single-view framework. The initial depth approximation *dz* plays a role in the convergence process. If they select an initial depth approximation of *dz* = 1 mm, which is actually the true simulated displacements from their computer simulations, then they obtain lower 3D root mean square errors (RMS) between optimized and true 3D coordinates. Depth initial estimates *dz* ∈ [1,2,3] mm produce RMS values less than 3 mm when using three consecutive monoplane images. The RMS values are accumulated values across the number of images used for the monoplane algorithm. The PA view simulations produce better results probably due to the projected helix points being distributed with no co-planarity in the images. On average, they are at most 2.074 mm from the true displacements when considering six consecutive images using approximate depth displacements of *dz* ∈ [1,2,3] mm. However, there is a trade-off with the accumulated 3D RMS errors being at most 8 mm when using the LAT view and six consecutive images. As expected, if initial depth estimates are far away from the true ground truth values, results deteriorate as seen when using *dz* ∈ [4, 5] mm.

**Table 1:** Computer simulation results for various depth estimations dz. (All values in mm).

	Left Lateral View				Antero-Posterior View			
*dz=0* #Images	mean	min	max	3D RMS	mean	min	max	3D RMS
3	2.188	0.148	3.840	3.976	1.033	0.096	1.917	1.911
4	1.752	0.094	3.837	4.527	0.976	0.003	2.067	2.613
5	1.463	0.003	3.951	5.152	0.929	0.007	2.128	3.204
6	1.150	0.015	3.373	5.324	0.869	0.004	2.160	3.674
*dz=1* #Images	mean	min	max	3D RMS	mean	min	max	3D RMS
3	1.491	0.043	2.877	2.763	0.578	0.005	0.929	0.983
4	1.272	0.039	2.876	3.122	0.651	0.006	1.080	1.541
5	1.247	0.101	2.998	3.802	0.657	0.010	1.133	2.004
6	1.230	0.011	2.413	4.411	0.641	0.002	1.165	2.398
*dz=2* #Images	mean	min	max	3D RMS	mean	min	max	3D RMS
3	1.254	0.005	1.913	2.048	0.915	0.060	1.830	1.742
4	1.223	0.098	2.628	2.813	0.977	0.002	1.996	2.592
5	1.363	0.006	3.052	4.095	1.045	0.003	2.070	3.443
6	1.522	0.021	3.327	5.645	1.114	0.001	2.109	4.314
*dz=3* #Images	mean	min	max	3D RMS	mean	min	max	3D RMS
3	1.190	0.059	2.860	2.341	1.890	1.050	2.794	3.142
4	1.418	0.008	3.643	3.873	1.942	0.896	2.954	4.458
5	1.730	0.009	4.072	5.787	2.004	0.842	3.023	5.815
6	2.074	0.012	4.351	8.097	2.074	0.825	3.057	7.220
*dz=4* #Images	mean	min	max	3D RMS	mean	min	max	3D RMS
3	1.698	0.014	3.862	3.390	2.866	2.040	3.758	4.635
4	2.197	0.007	4.659	5.567	2.913	1.885	3.912	6.471
5	2.568	0.064	5.091	8.037	2.974	1.832	3.976	8.363
6	3.021	0.465	5.375	10.979	3.042	1.820	4.006	10.314
*dz=5* #Images	mean	min	max	3D RMS	mean	min	max	3D RMS
3	2.668	0.978	4.862	4.714	3.842	3.030	4.723	6.154
4	3.173	0.969	5.673	7.476	3.885	2.873	4.872	8.528
5	3.544	0.812	6.111	10.492	3.944	2.822	4.930	10.965
6	4.004	1.424	6.399	14.028	4.011	2.814	4.955	13.468

### Left ventricle simulations: non rigid motion

**Figure 3:**
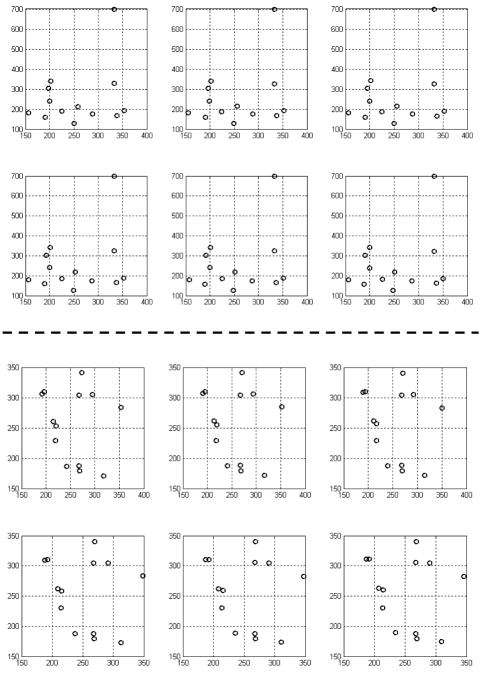
2D projections of 13 ventricle 3D crystal coordinates across six consecutive images representing (top) anterior/posterior and (bottom) left lateral X-ray gantry configurations.

The clinical data for the ventricle of a sheep was obtained from [10]. Figure 3 represents the 2D projections of six consecutive real time instants of the contracting ventricle using the previous gantry parameters for the helix simulations. They perform monoplane reconstruction across the six image frames for this type of non-rigid motion of the ventricle.

**Figure 4:**
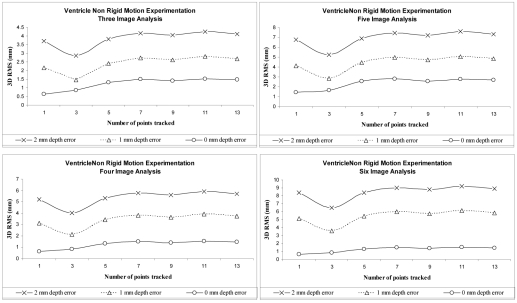
3D RMS Reconstruction Errors for Left Ventricle Analysis. Results for 13 markers on left ventricle undergoing non-rigid motion between three and six consecutive image frames.

Figure 4 shows the results obtained on the left ventricle data. The accuracy of depth determination will decrease if more than six C-arm images are used in their optimization scheme. In other words, monoplane reconstruction can still be achieved but with a higher RMS value. In such a case, the authors can compensate for this error by getting additional 3D information of the catheter at that particular time instant and then re-perform monoplane reconstruction and depth estimation in subsequent images using their equations. *A priori* information could also be obtained by CT for example, and the authors can easily obtain 3D coordinates of the catheter at the diastolic and systolic cardiac phases. Thus, using a C-arm fluoroscope and assuming 15 frames per second acquisition rate, they normally would have close to ten monoplane image frames representing the cardiac cycle. If they assume the 1^st^ frame would represent diastole and the last systole, they can estimate depth for the middle C-arm images by using the two *a priori* 3D representations obtained from CT, instead of using only a single *a priori* model as suggested in this paper. This protocol might alleviate failure to correctly estimate depth. The average 3D RMS increased from 2.5 mm to 5 mm when tracking 13 crystals across six images and using an initial guess of *dz* = 1 mm. Authors in [11], have demonstrated that point correspondence can be completed temporally to provide the minimal information required for robust 3D structure estimation using a total of 12 landmark points. From their monoplane analysis and Figure 4, they determine that a minimum of six tracked points begin yielding stable 3D RMS values. Worst case results show that when using a more realistic depth initial guess of *dz* = 2 mm, 3D RMS errors for the 13 crystals was about 8 mm with six consecutive C-arm images.

Here, 8 mm is a 3D accumulated error for all landmark points tracked across six consecutive single-view C-arm images. This means, it is the sum of all 3D errors for the 13 landmarks across the six images. In consequence, from prior work [3], the authors had estimated depth to within 1 cm of the true value using only 1 landmark, that is, the tip electrode on a single image. The algorithm does better; about 20%, as estimated depth to within 0.8 cm for 12 electrodes and for six images. Nevertheless, the accepted clinical threshold is set to 2 mm. Depending on the C-arm acquisition frame rate, six consecutive images probably depicts half of a cardiac cycle (i.e. between diastolic and systolic phases). This leads to the belief that additional constraints need to be added to the equations for future analysis, if the inherent non-rigid motion of the cardiac structures be recovered in its totality. This is determined by simply comparing the recovered 3D rigid RMS values in Table 1 as being more accurate than the non-rigid 3D values from Figure 4.

### Future work

The proposed algorithm and computer simulations demonstrated that rigid movements can indeed be recovered; hence to the reconstruction of rigid objects can certainly be attempted, such as catheter electrodes, across a monoplane sequence. Furthermore, the monoplane reconstruction procedure can be extended to clinical instruments such as arrhythmia ablation catheter tips as they are rigid objects as well.

## CONCLUSION

A new algorithm to estimate the depth of the mapping catheter tip was presented. The methodology exploits the spatio-temporal information of rigid structures in order to estimate their depth in the focal direction of the C-arm fluoroscope. Several conclusions can be made from the present work: (i) only C-arm images are used to detect 3D catheter positions with no added cost from expensive 3D mapping technologies, (ii) a 3D *a priori* model of the catheter is required at a first time instant to estimate subsequent depth positions at later image frames, (iii) a minimum of three consecutive C-arm fluoroscopy images are required to solve for rigid motion and interframe displacements of the mapping catheter, (iv) a minimum of six image points are required during the tracking phase in order to observe algorithm convergence and minimization of 3D RMS, and (v) non-rigid motion was not recovered as observed in the final reconstruction results when compared to the rigid simulations. This feasibility study provided results that were an improvement to those in the simple biplane projection method developed in [3] when using only a single image. The global objective remains to provide interventional assistance for cardiac ablation procedures. By exploiting spatial and projective information using only single-plane images, the authors aim to decrease overall intervention time and still maintain high-level accuracy when predicting the depth position of the mapping catheter. The ambition is to depict the ablation catheter electrodes in 3D accurately between the diastolic and systolic cardiac phases that, in turn, can help the interventionist during the ablation procedure. Future work will focus on adding non-rigid constraints to the monoplane equation, which capture the inherent shape of the rigid electrodes and not only their positions.
